# Analysis of Pull-out Tests for Resin-Grouted Rib Bolts

**DOI:** 10.1007/s42461-026-01619-8

**Published:** 2026-07-28

**Authors:** Khaled Mohamed, Yuting Xue, Dogukan Guner, Alper Kirmaci, Taghi Sherizadeh

**Affiliations:** 1 CDC NIOSH, Pittsburgh, PA, USA; 2 Missouri University of Science and Technology, Rolla, MO, USA

**Keywords:** Resin-grouted bolts, Rib bolts, Pull-out tests, Short encapsulation tests

## Abstract

Resin-grouted rib bolts serve as a crucial means of stabilizing spalling of coal ribs in underground coal mines. A comprehensive investigation into their efficacy was undertaken by a collaborative effort between the National Institute for Occupational Safety and Health (NIOSH) and Missouri University of Science and Technology (MST). This study involved pull-out tests of rib bolts in multiple locations, including six coal mines and the NIOSH research mine. A total of seventy-three (73) pull-out tests were conducted for rib bolts installed at different rotation speeds and a wide range of anchorage lengths, ranging from a short encapsulation length of 0.305 m to a fully grouted encapsulation length of 1.524 m. The test findings indicate that when short encapsulation bolts are installed at high rotation speeds, their anchorage capacity is significantly reduced, leading to failure at the bolt-grout interface. Conversely, when these bolts are installed at lower rotation speeds, they exhibit greater capacity, with failure occurring at a higher anchorage load equivalent to the yield load of steel rebar. Regardless of the rotational speeds used during installation of the fully grouted bolts, they consistently experienced failure at the ultimate load of the steel rebar. Most of the tests carried out on partially grouted bolts with anchorage lengths of 0.610–0.914 m have shown behavior patterns like the fully grouted bolts, although exhibiting reduced stiffness. The outcomes of this research offer a profound insight into the ways in which resin-grouted bolts enhance the stability of coal mine ribs.

## 1 Introduction

Coal ribs, which are the walls of coal pillars, are sometimes intentionally supported during mining operations to maintain their structural integrity. Due to the flexibility, effectiveness and relatively straightforward installation process, rib bolting systems have been widely used in underground coal mines. The installation of grouted bolts involves inserting a resin cartridge and bolt into a drilled borehole and driving the bolt to break and mix the resin to form a mechanical interlock between the bolt and surrounding rock strata. When coal ribs start to deform and dilate, load is induced in the bolt to reinforce the ribs. However, the rib bolting system is complex because it involves various components, namely bolt, grout and the surrounding rock, and their interactions. The properties of these components and their interaction potentially affect the overall performance of resin-grouted rib bolts. Due to the complexity, many different methods have been employed to investigate bolting behaviors through laboratory and field testing, theoretical analysis, and numerical simulation.

The occurrence of rib falls presents severe safety risks, encompassing injuries, fatalities, and equipment damage. The effectiveness of rib bolting systems depends on many factors, such as local geological conditions, the type and height of coal ribs, and stress on the ribs. Therefore, good rib support design must account for the impact of these factors. Inadequate support design could result in insufficient reinforcement and subsequent rib instabilities. The rib bolting design in U.S. underground coal mines currently relies on a trial-and-error approach. Researchers from the National Institute for Occupational Safety and Health (NIOSH) have been working on the development of -an engineering-based approach for coal rib stability analysis and bolting design [1, 2, 3].

**Fig. 1:**
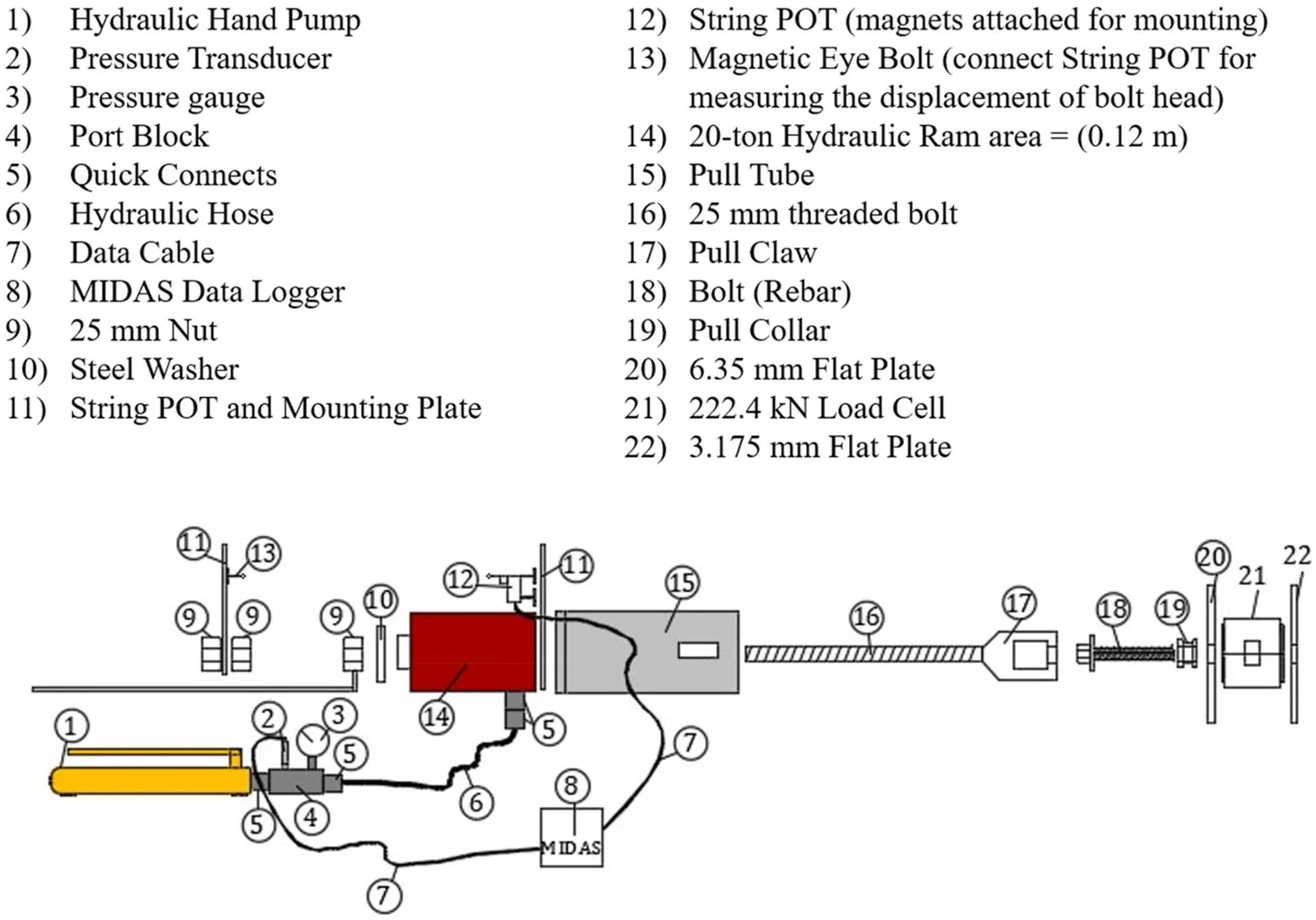
Pull-out test gear components

The pull-out test is a conventional method to investigate -the load transfer mechanism and anchoring capability of bolts, providing crucial information for engineering-based rib support design. It involves the gradual application of axial load using a hydraulic jack to measure the force required for the bolt to be pulled out. Pull-out tests can be conducted either in a laboratory or in the field. Figure 1 shows a schematic drawing of the components of the standard pull gear utilized in this study.

Laboratory tests are often favored over field tests due to their cost-effectiveness and ease of control [4, 5, 6, 7, 8, 9]. These laboratory experiments provide several advantages, enabling the examination of various factors affecting bolt performance. These factors encompass grout properties [10], bolt surface configurations [11, 12], bolt diameters [13], host rock properties [9], and confining pressure [14].

It is important to recognize that the bond characteristics observed in laboratory tests may not accurately represent the conditions of bolts in the field. These- laboratory tests do- not account for various- factors, such as rock mass quality, and machinery effects, all of which have the potential to impact bolt performance. In a comprehensive study conducted by Cincilla [15] in 1986, over 1,000 pull-out tests were carried out on roof bolts across 11 underground coal mines throughout the United States. These tests encompassed various anchorage lengths of 0.305, 0.457, 0.610, and 1.219 m. The findings revealed a repeated trend: in most of the roof bolt tests, the behavior of the steel rebar became the dominant factor in the bolt’s response to axial loading conditions imposed during the pull-out test, typically once the resin grout length exceeded approximately 0.610 m. To further investigate the anchorage failure of roof bolts, Mark et al. [16] conducted Short Encapsulation Pull Tests (SEPT) in the roofs of underground coal mines. For these tests, the researchers grouted only the top 0.305 m of the bolt so that the bolts would not reach yielding during the tests. Subsequently, Chugh et al. [17] carried out short encapsulation pull tests (SEPTs) to establish a comprehensive database for mine roofs in the Illinois Coal Basin. Their dataset comprised data from over 200 tests conducted in 20 mines across Illinois, Indiana, and Western Kentucky, encompassing a broad range of overburden depths, bolt lengths, and bolt diameters. The SEPTs conducted by Aziz et al. [18] further confirmed that an encapsulation length of 0.305 m is appropriate for pull-out tests aimed at investigating roof bolt anchorage failure. Extending the encapsulation length beyond this point was found to potentially induce bolt yielding.

However, it is important to highlight that there is a significant lack of pull-out tests performed on the rib bolts, indicating the need for additional research in this specific area. The objective of this study is to investigate the effectiveness of rib bolting in various coal seams within the Eastern mines of the United States. This will be accomplished by performing a series of pull-out tests in those mines in addition to the NIOSH Safety Research Mine. The in-situ pull-out tests conducted on rib bolts in this study will address the existing gap in the limited availability of such tests, providing valuable data for researchers focused on understanding the support mechanisms of rib bolts in coal mines.

## 2 In-Situ Pull-Out Tests for Resin-Grouted Rib Bolts

A total of 73 pull-out tests were carried out across six different coal mines and at the NIOSH Safety Research Mine. The rib bolts were tested in five different coal seams: Pittsburgh Seam, No. 2 Gas Seam, Kellioka Seam, Jawbone Seam, and Pocahontas No. 3 Seam. No. 5 bolts, which have a diameter of 15.875 mm, were dominantly tested in this study, although a limited number of No. 6 bolts with a diameter of 19.05 mm were also tested. All the tested bolts were grade 60 with a tensile strength of 414 MPa. The yield loads for No. 5 and No. 6 bolts are 82 kN and 118 kN, respectively. Most of the tested bolts were 1.219-m long, although some were 1.524 m in length. All the bolts tested were installed in holes with a diameter of 25.4 mm.

The performances of rib bolts with three different anchorage types were examined in this study:

**(1)** fifty-one (51) pull-out tests were conducted on short encapsulation (SE) bolts, each having an anchorage length of 0.305 m, **(2)** fifteen (15) pull-out tests on fully grouted (FG) bolts, and **(3)** seven (7) pull-out tests on partially grouted (PG) bolts with anchorage lengths ranging from 0.610 m to 0.914 m.

All the rib bolts were installed within the coal seams. Most of the bolts were installed horizontally, although there were a few exceptions where the installation deviated from the horizontal orientation. In this study, we did not select the resin type for pull-out tests. Instead, we used the resin cartridge provided by the mine for rib bolting. The spin time for all tested bolts was approximately 4–10 s, with a hold time of around 60 s. A minimum of 2 h elapsed between bolt installation and testing. It is important to highlight that different roof bolters and operators participated in the installation of rib bolts in this study. The speed of bolt installation was generally unregulated, except when tests were performed in the NIOSH Research Mine.

During the pull-out tests, the bolts were subjected to a minimum axial displacement of 20 mm at the bolt head. In the process of conducting pull-out tests on rib bolts, our primary goal was to prevent exceeding the fracture load of the steel rebar, except in a few cases where the intention was to apply force until the bolt fractured, while adhering to all essential safety precautions.

### 2.1 Short Encapsulation Pull-Out Tests

**Fig. 2:**
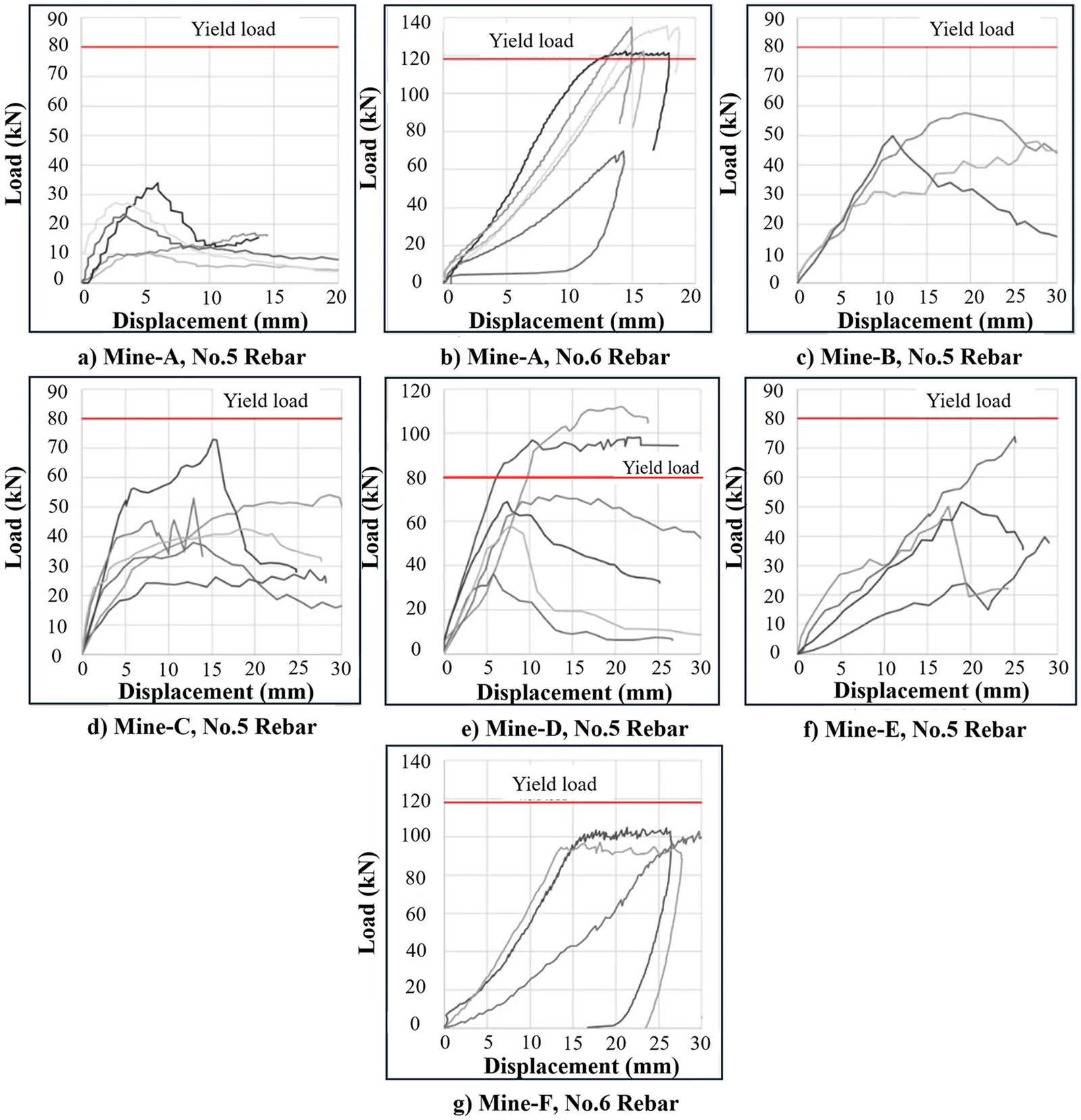
Load-displacement curves for SEPTs

To assess the anchorage capacity of grout while avoiding the yielding of steel rebar, the anchorage length for all SEPTs was set to 0.305 m as established in previous research studies [16, 17, 18]. Figure 2 displays the load-displacement curves obtained from the SEPTs conducted in six different mines. The y-axis represents the recorded load in kN, while the x-axis represents the measured head displacement in mm. The red line indicates the yield load of the examined bolts in kN. It is worth mentioning that the rib bolts No. 5 and No. 6 in Mine-A for SEPTs were installed on different days by different operators. Additionally, the tests in Mine-E were intentionally terminated before reaching bolt yielding because of inadequate safety precautions in the event of bolt failures.

It is apparent from Fig. 2 that no unique load-displacement pattern can be extracted from the conducted SEPTs. These tests exhibited a range of behaviors, with some SEPTs demonstrating softening characteristics, while others exhibited consistent plastic behavior. Table 1 provides a summary of the key features extracted from Fig. 2 for all the SEPTs. Notably, Table 1 shows a significant variation in the measured average peak loads for No. 5 bolts, ranging from 26% to 88% of the yield load of steel rebar. Conversely, No. 6 bolts exhibit consistent average peak loads, ranging between 83% and 74% of the yield load of steel rebar.

Prior to carrying out the SEPTs, there was no expectation that any of the tested bolts would yield and that failure would happen at the grout/bolt interface. However, the tests revealed that in many of the performed SEPTs, bolt failure was the result of steel yielding. Figure 2b shows that in Mine-A the steel rebar yielded in all the SEPTs for No. 6 bolts, except for one test. Also, Fig. 2e shows that some SEPTs encountered yielding of steel rebars and many of the tests achieved loads of 75% of the yield load. Figure 2f illustrates that in Mine-E, the peak loads of the SEPTs reached 83% of the yield load, with one test intentionally terminated before reaching the point of bolt yielding.

**Fig. 3:**
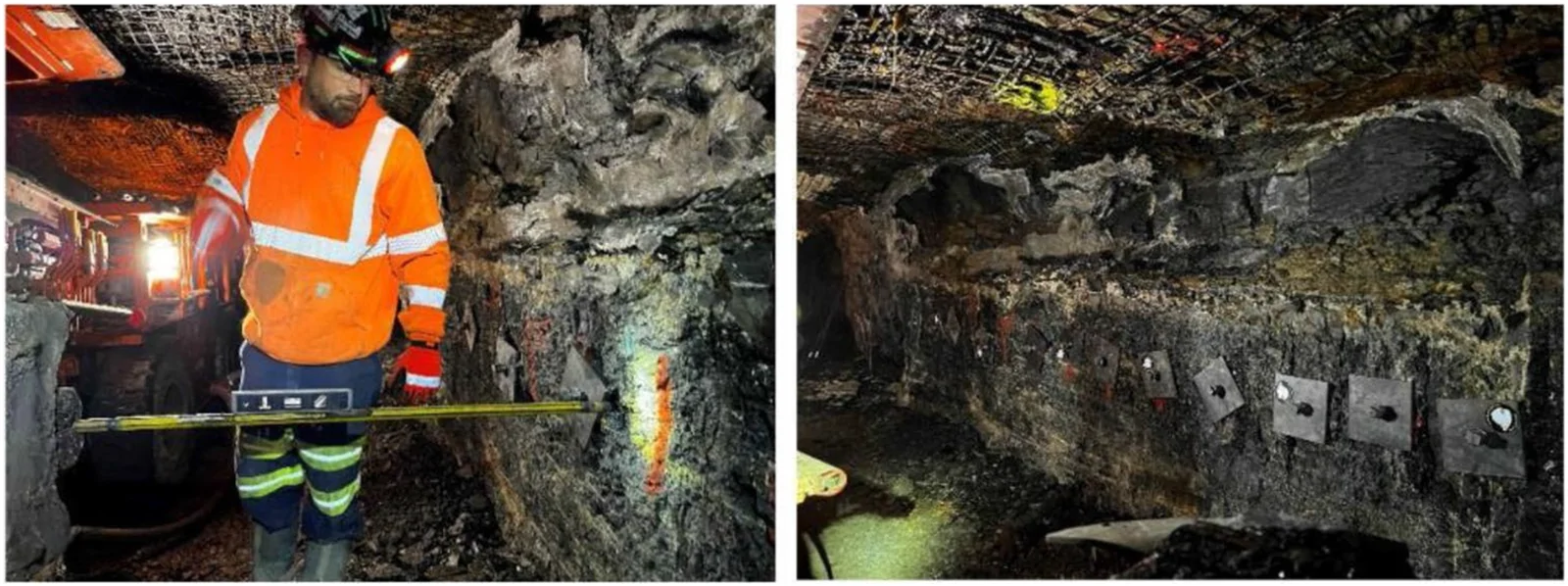
SEPTs preparation carried out at the NIOSH Safety Research Mine

Could the larger bolt diameter tested in Mine-A be a contributing factor for achieving the yield load in SEPTs, as illustrated in Fig. 2b? Could the rotational speed employed during bolt installation have an impact on achieving the yield load in some of the SEPTs? To address the preceding questions, more controllable SEPTs were carried out in the NIOSH Safety Research Mine. These experiments included the evaluation of two bolt sizes, No. 5 and No. 6. Additionally, two different levels of rotation speeds were used for bolt installation: 170 rpm and 780 rpm. Figure 3 displays images illustrating the process of drilling a horizontal hole in an almost vertical coal pillar rib, along with a photo of the bolts successfully installed within the coal seam.

**Fig. 4:**
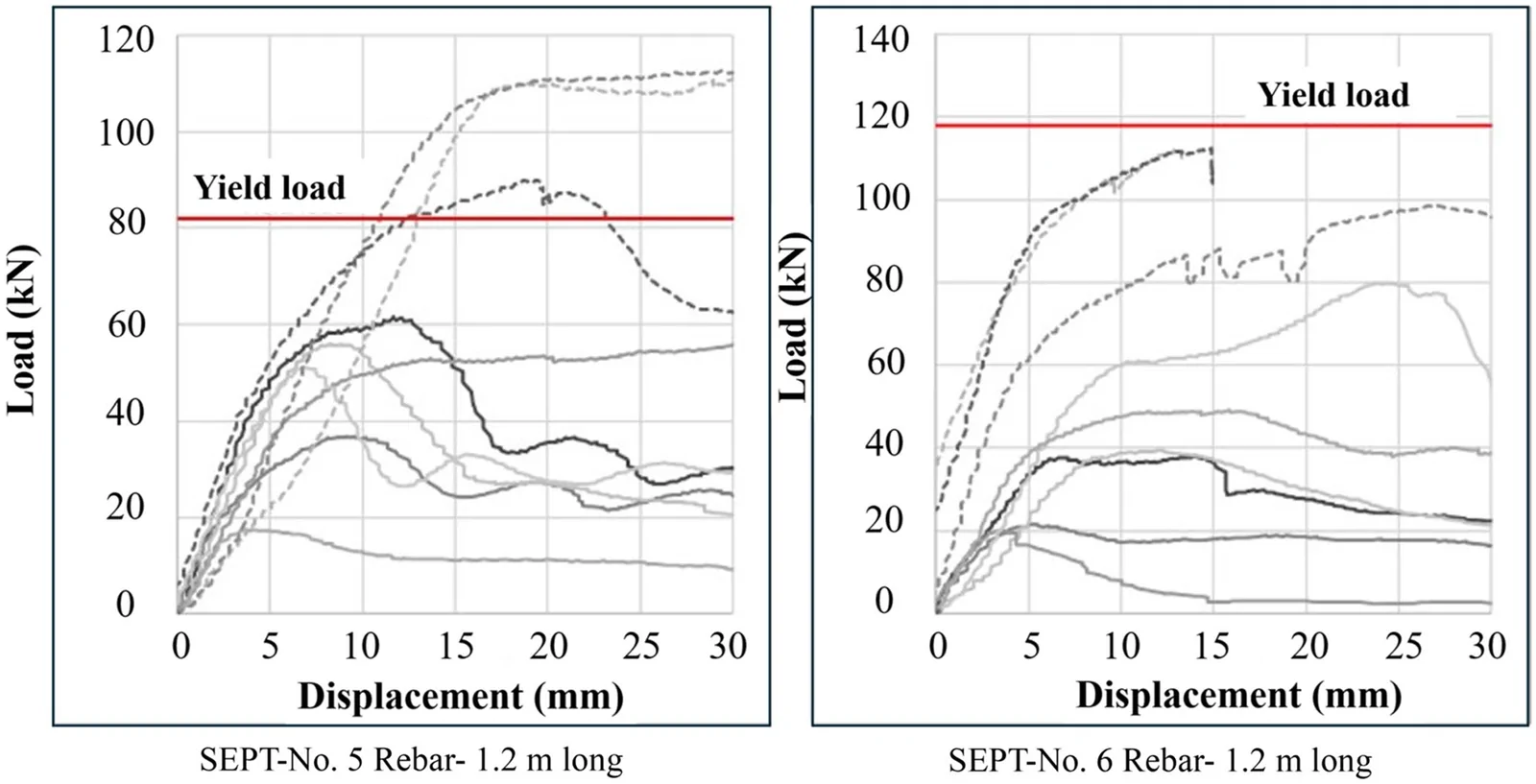
Load-displacement curves for SEPTs conducted in the NIOSH Safety Research Mine. Solid curves represent bolts installed at a high rotation speed of 780 rpm. Dotted curves represent bolts installed at a low rotation speed of 170 rpm

Figure 4 presents load-displacement curves resulting from all SEPTs conducted at the NIOSH Safety Research Mine. The solid lines represent the load-displacement curves of bolts installed at a high rotational speed of 780 rpm, while the dashed lines show the load-displacement curves for bolts installed at a low rotational speed of 170 rpm. Table 1 provides a summary of the key features of all SEPTs extracted from Fig. 4.

Figure 4 clearly demonstrates the profound influence of the rotation speed utilized during bolt installation on the measured anchorage capacity of SEPTs. Employing a high rotational speed during bolt installation and spin time of approximately 4–10 s results in diminished anchorage capacities. A comparable trend was previously observed by Mishra [19], who conducted laboratory-based SEPTs to investigate the impact of spin time and rotational speed on bolt performance. These findings emphasize the significance of both spin time and rotational speed in determining bolt capacity.

**Fig. 5:**
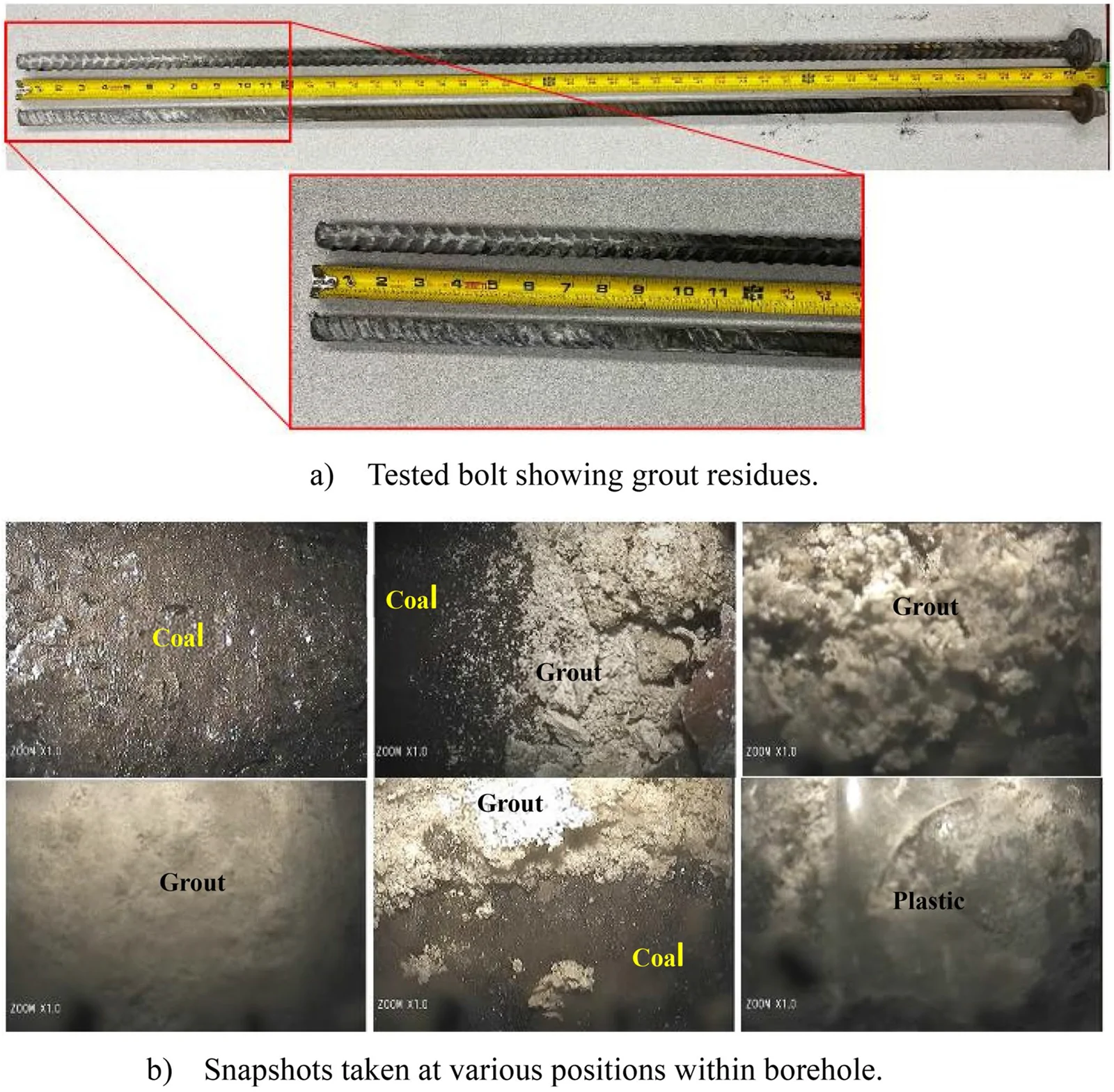
Post-testing photographs showing No. 5 tested bolt installed at a rotational speed of 780 rpm, along with snapshots captured within the drilled borehole

Bolts installed at high rotation speeds were fully extracted from the holes. Figure 5 shows post-testing images of a bolt installed at a high rotational speed, along with snapshots taken inside the drilled borehole. These images reveal that the bolt/grout interface of the No. 5 tested bolt exhibits signs of damage, while the grout appears to be firmly bonded to the borehole boundaries.

The SEPTs conducted in the NIOSH Safety Research Mine led to the conclusion that if an optimal rotational speed is employed during bolt installation, it is likely that the anchorage capacity of rib bolts, as measured by SEPT, would be equivalent to the yield load of steel rebar, irrespective of the bolt diameter. The quality of grouted bolts is not solely determined by a single parameter. Rather, it depends on a complex interaction of various factors. These factors include resin speed, applied thrust and torque, as well as the mixing time. Excessive mixing can have a detrimental effect on anchorage quality, as illustrated in Fig. 5b. Further experiments are required to establish the rotation speed and the mixing time thresholds beyond the manufacturer’s recommendations that can adversely affect the quality of fully grouted bolts. The controlled SEPTs conducted at the NIOSH Safety Research Mine were designed to replicate field installation practices where higher rotational speeds are accompanied by similar spin times. Under these conditions, higher rotational speeds resulted in a significantly larger number of revolutions, which in turn produced excessive mixing and degradation of the bolt–grout interface. This interpretation is supported by the post-test observations (Fig. 5), where bolts installed at 780 rpm exhibited damaged bolt–grout interfaces while the grout remained well bonded to the borehole wall.

### 2.2 Partially Grouted Pull-Out Tests

Following the same procedure adopted for conducting SEPTs, partially grouted pull-out tests (PGPTs) for rib bolts were tested in two mines. Unfortunately, the rotation speed of the roof bolter was not recorded in those tests. In Mine-B, two anchorage lengths (0.61 m and 0.914 m) were tested for the No. 5 bolt with a bolt length of 1.219 m.

**Fig. 6:**
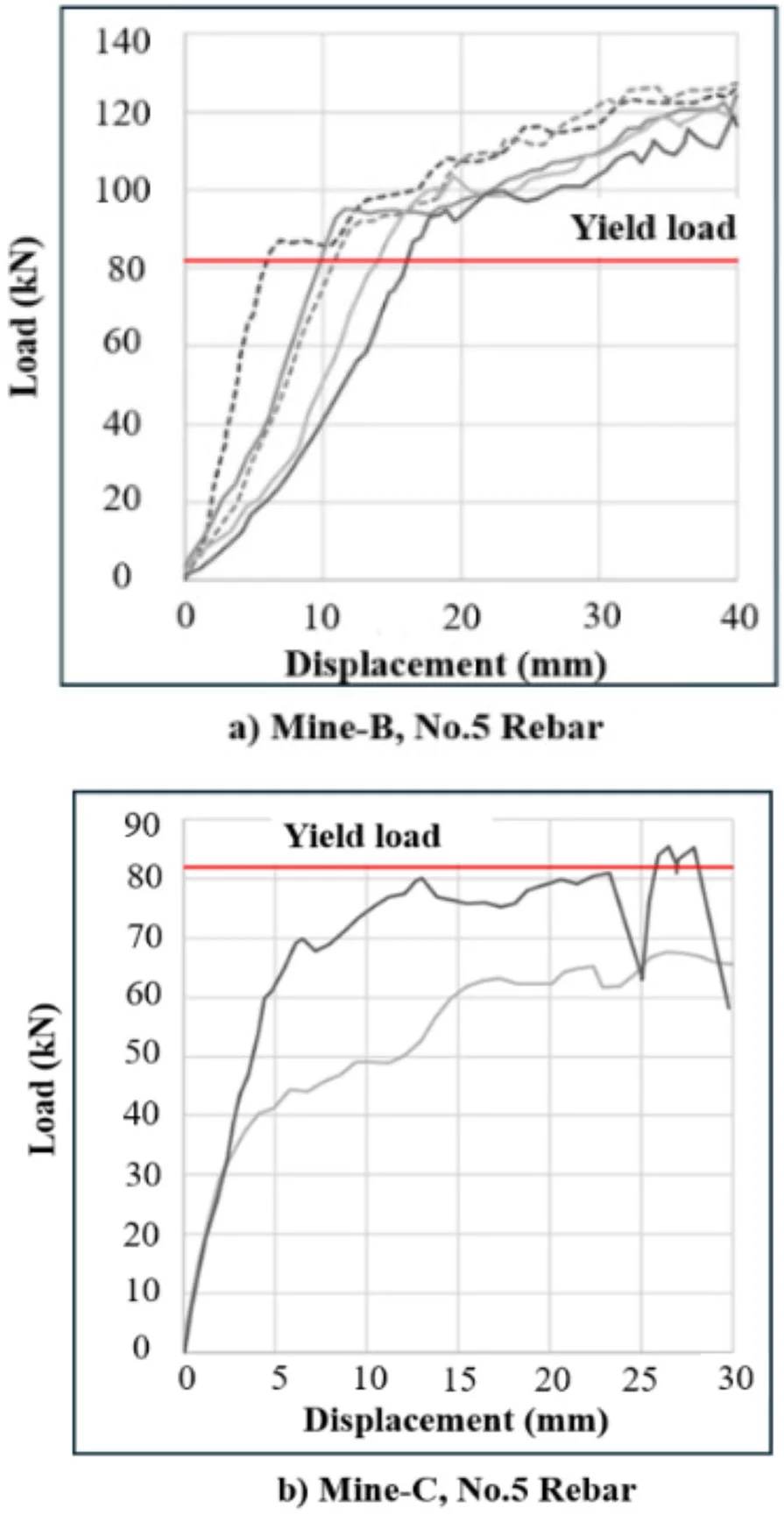
Load-displacement curves of PGPTs with solid lines denoting a 0.61-m anchorage length and dashed lines indicating a 0.914-m anchorage length

Figure 6a shows the load-displacement curves of PGPTs in Mine-B. It shows that all PGPTs experience steel rebar yielding with stiffer load-displacement curves for a longer anchorage length of 0.914 m. Figure 6b illustrates the load-displacement curves of PGPTs carried out in Mine-C. In tests conducted in Mine-C, the yielding capacity of steel rebar was reached in one of the tests, while in the other test, the peak load reached approximately 83% of the yield capacity of a steel rebar.

Table 2 summarizes the key features of all conducted PGPTs extracted from Figure 6. The anchorage capacity of rib bolts, as determined by PGPTs, primarily relies on the yielding of the steel rebar, irrespective of unregulated rotation speeds adopted during their installation.

### 2.3 Fully Grouted Pull-out Tests

**Fig. 7:**
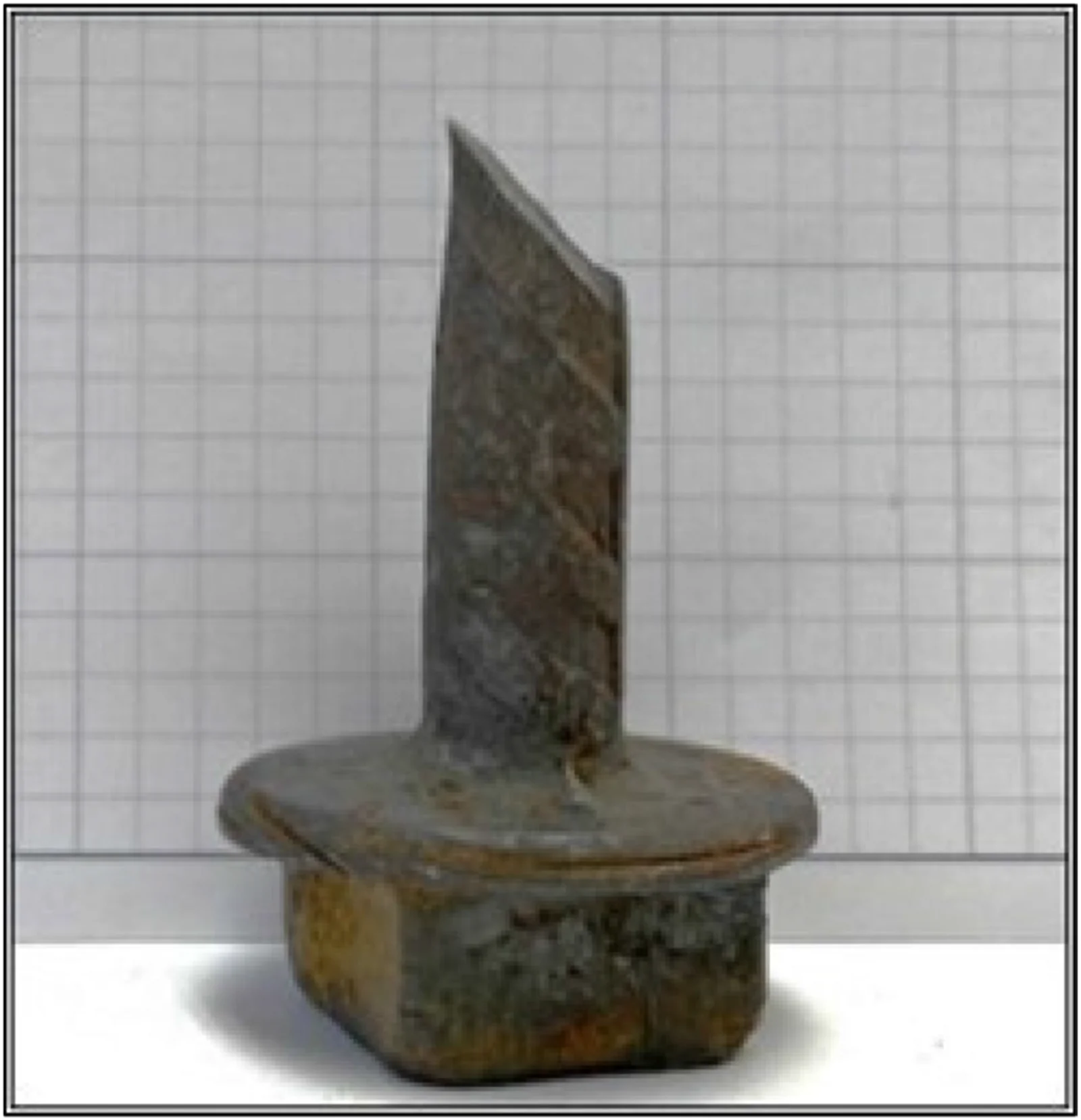
Head portion of fractured bolt showing necking behavior induced by fully grouted pull-out tests (FGPT)

**Fig. 8:**
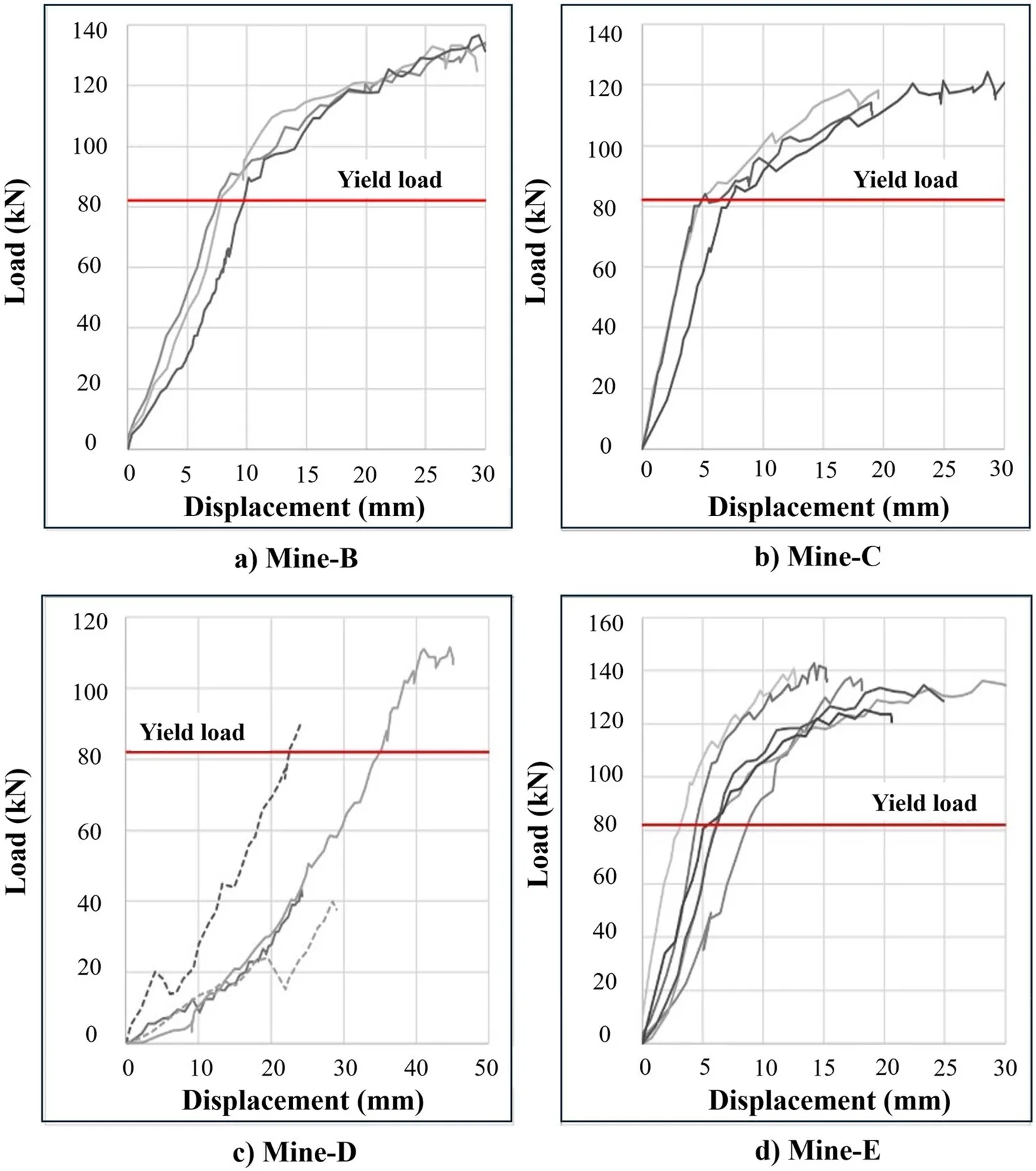
Load-displacement curves for FGPTs, where solid lines correspond to a 1.219-m bolt length and dotted lines correspond to a 1.524-m bolt length

Pull-out tests were carried out on fully grouted bolts in four mines. The rotation speed during the bolt installation was not regulated in all the FGPTs. These tests were conducted until the bolts failed for some tests with strict adherence to safety protocols throughout the testing process. Figure 7 shows the head portion of one of the fractured bolts, showing the necking behavior induced during the pull-out test. Figure 8 illustrates the load-displacement curves of all FGPTs. Table 3 lists a summary of the key features of all conducted FGPTs as extracted from Fig. 8. As anticipated, the load-bearing capacity of rib bolts within all FGPTs primarily relies on the yielding characteristics of the steel rebars, regardless of the unregulated rotation speed employed during their installation.

In a prior investigation focused on the performance of mechanically anchored rib bolts within underground coal mines, significant findings highlighted the substantial influence of coal mass strength on the observed bolt capacity [3]. In the ongoing research, we initially hypothesized a similar influence of coal mass strength on the measured capacity of resin-grouted bolts. However, our findings did not support this hypothesis. Instead, we found that coal mass strength could impact the measured stiffness of grouted bolts, with stronger coal seams exhibiting a stiffer bolt behavior. This observation was quite evident in Mine-E when rib bolts were tested within the soft Pocahontas No. 3 coal seam.

## 3 Summary

Coal rib bolting systems are essential for ensuring the stability of underground coal mines. Poorly designed rib bolts can result in instability, leading to significant safety hazards. To address this issue, NIOSH researchers are developing an engineering-based approach to improve the reliability of rib bolting designs. Their study involved 73 in-situ pull-out tests conducted on rib bolts across various coal mines in the eastern U.S. These tests were performed on different coal seams and focused on grade 60 bolts, which have a tensile strength of 414 MPa. The study primarily involved No. 5 bolts with a 15.875-mm diameter and examined three anchorage types: short encapsulated, fully grouted, and partially grouted bolts, with varying anchorage lengths.

To assess grout anchorage capacity while preventing steel rebar yielding, SEPTs with a fixed 0.305-m anchorage length were conducted, and results from SEPTs in various mines indicated diverse load-displacement patterns. Notably, No. 5 bolts exhibited a wide range of peak loads, while No. 6 bolts were more consistent. Unexpectedly, some SEPTs experienced steel yielding. To investigate these issues, controlled SEPTs were performed at the NIOSH Safety Research Mine, testing two bolt sizes and two rotation speeds (170 rpm and 780 rpm). The controlled SEPTs illustrate how rotational speed and number of rotations during bolt installation profoundly influence SEPT anchorage capacity, with higher speeds and number of rotations resulting in reduced anchorage capacity. The controlled SEPTs showed that using higher rotational speeds, which led to more revolutions, causing excessive mixing and damage to the bolt–grout interface, as confirmed by post-test observations where bolts installed at 780 rpm showed interface damage despite good grout bonding to the borehole wall.

Both the PG and FG pull-out tests revealed that the anchoring strength of the tested bolts depends on the yielding of the steel rebar. Consistent loading patterns were observed in both PG and FG pull-out tests, regardless of the rotation speed used during bolt installation.

In this study, pull-out tests were carried out on rib bolts within several eastern coal mines. The research focused on the performance of Grade 60 steel rebars, specifically No. 5 and No. 6. The conclusions derived from the experimental results are limited to the mechanical properties of the coal seams tested and the properties of the bolts that were used. A more comprehensive study would have to be conducted to make any conclusions on the overall contribution of rib bolts to entry stability.

## Figures and Tables

**Table 1:** Summary of the key features of all SEPTs

Mine	Coal seam	Number of tests	Rebar diameter (mm)	Bolt length (m)	Average peak load (kN)	Load range (kN)	Average peak displacement (mm)	Rotation speed (rpm)
A	No 2 Gas	5	19.05	1.219	115	69-134	15	n/a
		5	15.875	1.219	22	10-34	6	n/a
B	Pittsburgh	3	15.875	1.219	52	48-58	19	n/a
C	Pittsburgh	6	15.875	1.219	47	24-72	17	n/a
D	Kellioka	7	15.875	1.219	72	36-111	9	n/a
E	Pocahontas	2	15.875	1.219	45	40-50	23	n/a
	No. 3	2	15.875	1.524	63	52-73	22	n/a
F	Jawbone	3	19.05	1.524	98	93-101	20	n/a
NIOSH Safety Research Mine	Pittsburgh	3	19.05	1.219	107	98-112	19	170
		6	19.05	1.219	79	17-61	25	780
		3	15.875	1.219	103	90-109	18	170
		6	15.875	1.219	46	17-61	9	780

**Table 2:** Key features of PGPTs for bolts with a length of 1.219 m and rebar diameter of 15.875 mm

Mine	Coal seam	Number of tests	Anchorage length (m)	Average 1^st^ peak load (kN)	1^st^ peak load range (kN)	Average 1^st^ peak displacement (mm)
B	Pittsburgh	3	0.610	98	95-103	17
		2	0.914	92	87-92	10
C	Pittsburgh	2	0.686	59	40-77	6

**Table 3:** Key features of FGPTs for bolts with rebar diameter of 15.875 mm

Mine	Coal seam	Number of tests	Bolt length (m)	Average 1^st^ peak load (kN)	1^st^ peak Load range (kN)	Average 1^st^ peak displacement (mm)
B	Pittsburgh	3	1.219	94	89-96	10
C	Pittsburgh	2	1.219	85	84-87	6
D	Kellioka	6	1.219	95	81-106	7
E	Pocahontas No. 3	2	1.219	n/a	n/a	n/a
		2	1.524	110	n/a	41

## Data Availability

The data that support the findings of this study are available from the corresponding author upon reasonable request.
